# A Conversation with Noah Whiteman

**DOI:** 10.1021/acscentsci.4c00431

**Published:** 2024-03-29

**Authors:** Carolyn Wilke

As living chemical factories, plants pump out compounds that people have used for centuries, from the aspirin in willow bark to the isothiocyanates in wasabi. “These things that show up in our food and medicine—none of it evolved for us,” says Noah Whiteman, an evolutionary biologist at the University of California, Berkeley, and author of the new book *Most Delicious Poison*.

**Figure:**
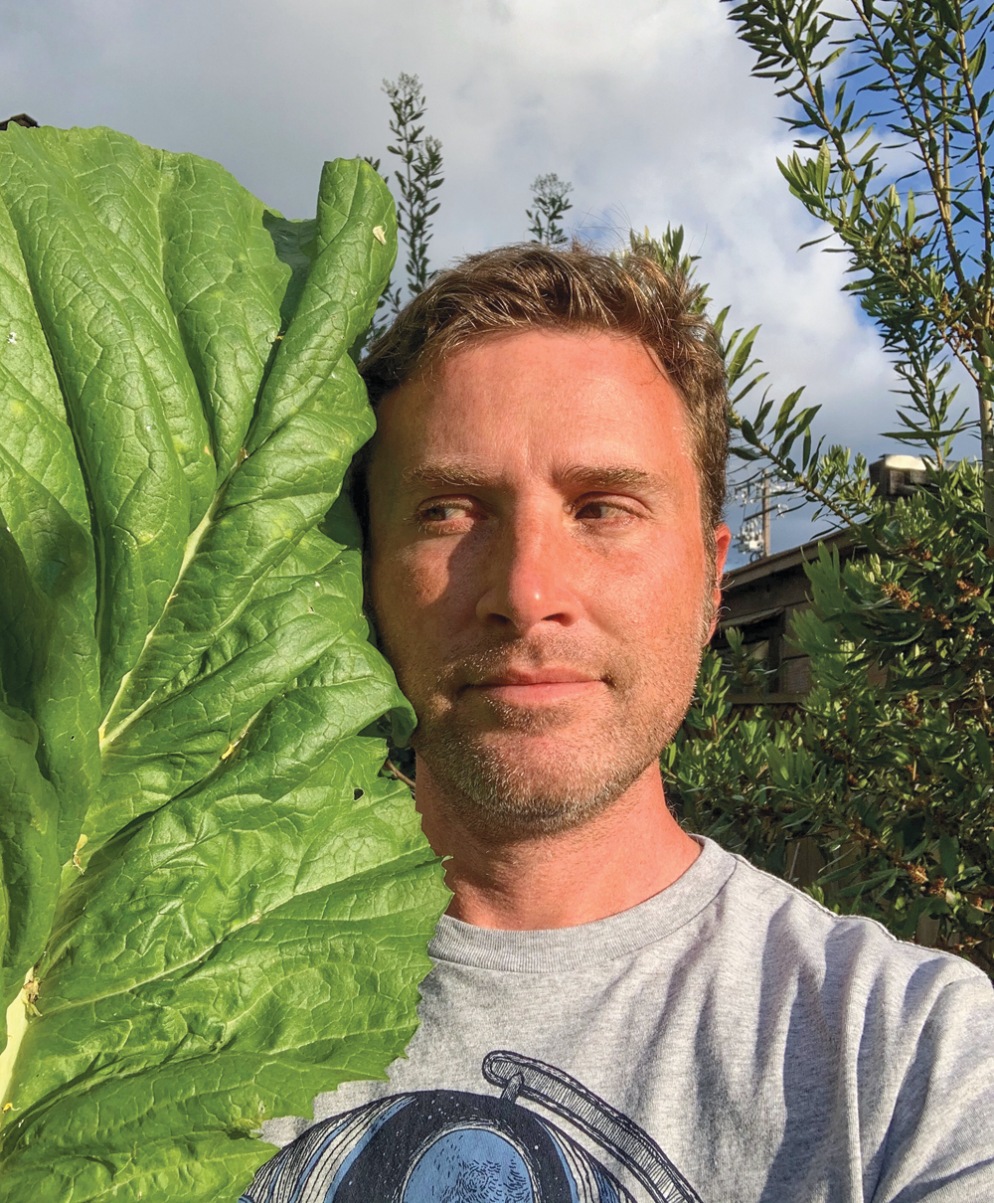
Noah Whiteman grows rhubarb in his poison garden. Rhubarb leaves contain oxalate and alkaloids that make them quite toxic—so do not eat the leaves, Whiteman cautions. Photo courtesy of Noah Whiteman.

Many of these chemicals evolved as toxins that plants use to battle the animals seeking to eat them. In his book, Whiteman explores the vast landscape of plant toxins that people consume, examining their ecology and tracing their adoption by humans, including Indigenous peoples worldwide. As Whiteman illustrates the interplay of biology, chemistry, and human culture, it becomes apparent that chemists can find insights from the plants and environments that gave rise to the compounds that tickle our taste buds and treat our diseases.

Carolyn Wilke spoke with Whiteman about the chemistry of nature’s poisons and their influence on human culture and genetics. (This interview was edited for length and clarity.)

## What are some examples of the plant poisons that lurk in our refrigerators, spice racks, and gardens?

Nicotine and caffeine are two daily use stimulants, and both are alkaloids that affect our nervous systems. And then a whole set of molecules—that are often phenols, terpenoids, or alkaloids—that are in other spices, ranging from things like mint, which has menthol, to oregano, to thyme, which has things like thymol.

One that is lurking in citrus, and in things in the parsley or dill family, is furanocoumarins. Grapefruit juice has furanocoumarins that interact with our metabolism to detoxify drugs. They target a specific enzyme, and the drug doesn’t get eliminated from our bodies at the rate that has been studied in clinical trials. So those levels can go up [to unsafe levels] over a period of hours or days if you continue to drink grapefruit juice.

## What drew you to learn about the chemistry of plants’ toxins and their evolution?

I probably first encountered this idea when my dad showed me that milkweed produces a milky sap. He said, basically, don’t touch or drink that sap—it’s poisonous. But then I saw these zebra-striped monarch caterpillars feeding on these beautiful plants. Later, in my lab, we started studying how insects use plant toxins as defenses of their own, like how the monarchs get cardiac glycosides from the milkweeds.

**Figure:**
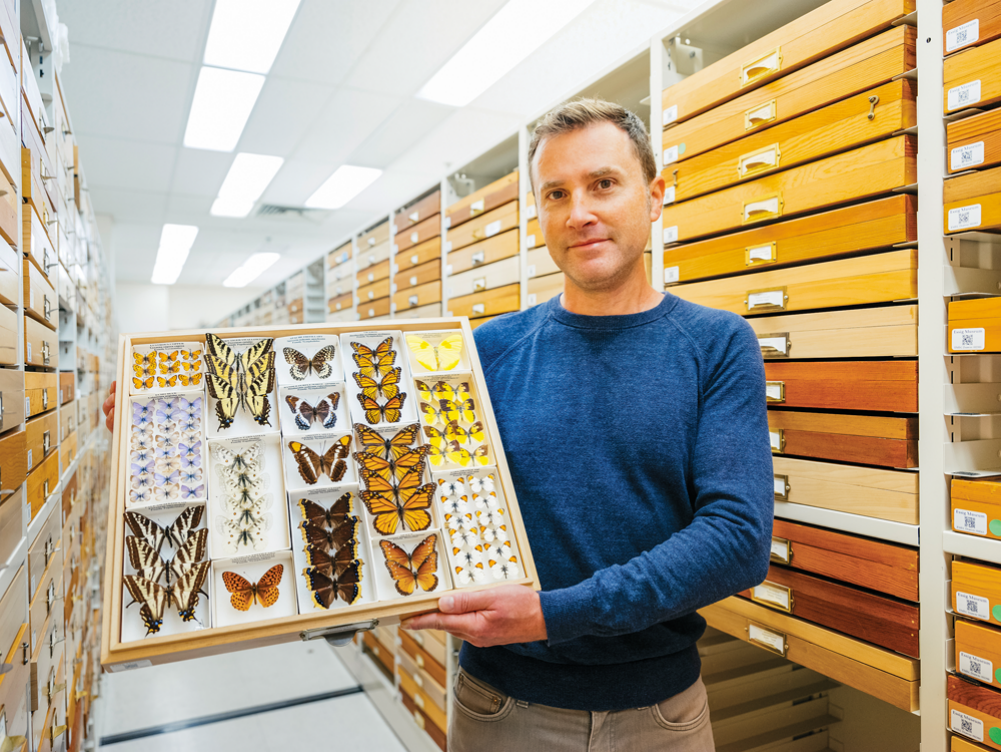
Noah Whiteman has studied how some insects, including monarch butterflies, collect toxins from the plants they eat to make themselves toxic to predators. Credit: Elena Zhukova.

## How do we know that so many key compounds in our foods and spices evolved through an arms race between plants and the pests that prey on them?

These are chemicals that are often bitter, spicy, painful—but also have some molecular function of targeting some animal system, whether the nervous, digestive, or muscular system. These chemicals are costly to make, and they only provide an advantage in the presence of enemies. That’s sort of proof that they are evolved and maintained as defenses primarily.

We share this basic biology with these other organisms; the chemicals often target the same things in us and insects. The thing that’s fascinating is that through human culture, which is coevolving with our genes, we’ve evolved the ability to tap into this reservoir [in beneficial ways] as some insects have.

## What is an example of how human culture and genetics have evolved with these compounds?

Favism is a hereditary disorder caused by a mutation in the *G6PD* gene. That mutation makes it difficult for red blood cells to control their redox environment. People with favism get anemia when they eat fava beans, strangely—that’s why it’s called favism.

Fava beans, also called broad beans, contain vicine alkaloids. People who don’t carry mutations that cause favism easily deal with these alkaloids that get into the blood.

But people with favism—the most common hereditary disorder in humans—are more resistant to malaria. It doesn’t seem to be a coincidence. Researchers have looked at where people consume fava beans, which includes Southeast Asia, for example, and they have *G6PD* mutations. The idea is that these vicine alkaloids cause anemic episodes that remove blood cells that could be infected by malarial parasites from the body.

## And then there are plants that are sources of molecules for pharmaceuticals. How have these shown up in our lives? In the book, you talk about the development of the birth control pill.

This professor, Russell Marker at [Pennsylvania State University], isolated the chemical diosgenin from a beautiful plant called trillium at a time when people wanted to synthesize hormones—progesterone, estrogen, testosterone, cortisol, and cortisone. Up to that point, the only way to get them was to isolate them from the animal glands. It was onerous and not sustainable.

But plants make steroid-like molecules, like diosgenin, that have a similar backbone. Marker quit his job to search for sources bigger than trillium plants. He drove to the U.S. Southwest and found plants in the yam family that produced giant tubers. Then he went into Mexico and found bigger ones. Some weighed like 200 pounds [90 kg].

Marker started this company in Mexico City called Syntex, and there, this undergraduate student, Luis Miramontes, synthesized the first semisynthetic progesterone, called norethindrone, one of the molecules that [would come to be] used in the birth control pill. That’s an amazing story, but why would the tubers be making this? We don’t know. Diosgenin is a saponin. [Saponins] may be an insect growth regulator because they’re similar to hormones, but a better hypothesis is that they disrupt the cell membrane, and so they’re toxins to insects.

**Figure:**
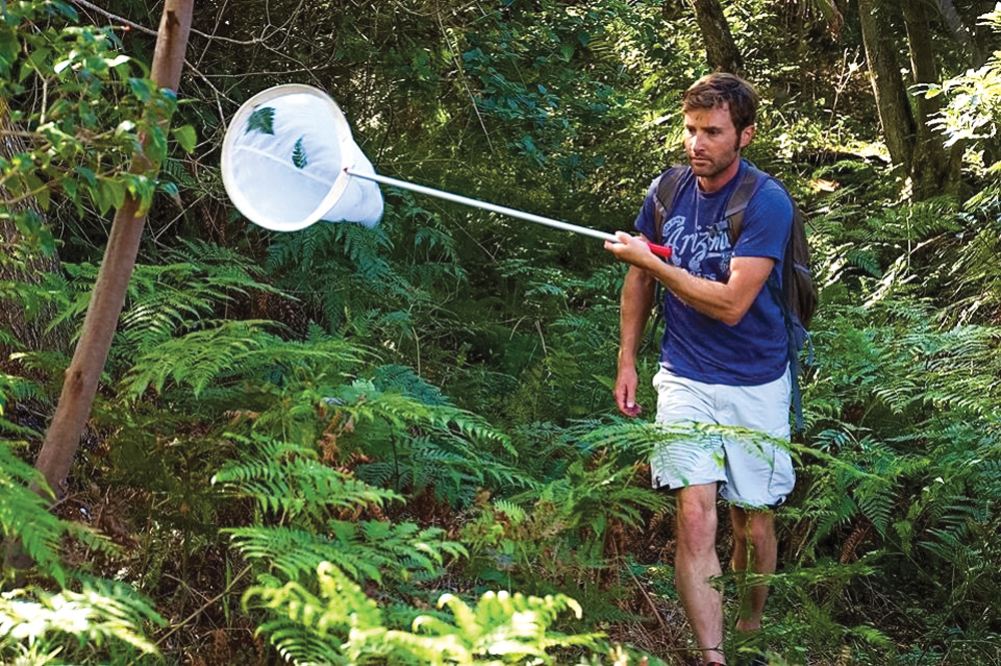
Noah Whiteman collects plant-munching flies that attack toxic bracken fern. Credit: Nathalie Nagalingum.

## What can chemists—for instance, those working in the field of natural products research—learn from evolutionary biologists?

One of the things that chemists can learn is to step back and ask themselves why these chemicals evolved. That can provide insight into the function of these things—the ultimate purpose that they serve for the organism that is making them.

The full story is the life cycle of that molecule—how it’s made, where the plant puts it in its cells or tissues, what happens when an animal eats it. Does it matter if it’s a caterpillar, deer, or human? Does the pH of the gut matter? If you don’t have information like that, you’ll miss a dimension to the bioactivity of the molecule.

## What’s the fate of nature’s chemical trove under climate change?

The vast majority of plant toxins are in the most endangered places in the world and are on lands with peoples whose cultures and identities are the most threatened. These are in the tropics, on Indigenous lands, and the plants are best known by Indigenous people.

Those places and people should be protected for their own sake. But if we conserve the tropics and empower the people who live there, so many problems—carbon sequestration, biodiversity loss—will be solved in addition to it just being the right thing to do.

## Carolyn Wilke is a freelance contributor to

Chemical & Engineering News,*the independent news outlet of the American Chemical Society*.

